# Acute Mesenteric Ischemia: Preexisting Comorbidity Determines Short-Term Outcome and Quality of Life in Long-Term Survivors

**DOI:** 10.1159/000526921

**Published:** 2022-11-24

**Authors:** Maria Witte, Manuela Neese, Matthias Leuchter, Mark Philipp, Ernst Klar, Clemens Schafmayer

**Affiliations:** Department of General, Visceral, Vascular, Thoracic and Transplant Surgery, University Medical Center Rostock, Rostock, Germany

**Keywords:** Acute mesenteric ischemia, Quality of life, EQ-5D, Charlson comorbidity index, Long-term outcome

## Abstract

**Introduction:**

Acute mesenteric ischemia (AMI), either arterial or venous, is still a devastating disease with poor prognosis. It is unknown, whether AMI is associated with impaired quality of life (QoL) in long-term survivors.

**Material and Methods:**

This retrospective analysis includes 64 patients with occlusive arterial or venous mesenteric ischemia treated operatively between 2008 and 2016 at the University Medical Center Rostock. Short-term outcome with focus on comorbidities was measured by the Charlson comorbidity index (CCI) an instrument that operationally measures comorbidity based on 17 clinical parameters including age. Operative outcome in view of enterostomy placement and long-term outcome measured as QoL by the EQ-5D in the long-term survivors were evaluated. The EQ-5D is a standardized, self-reported five-dimension QoL questionnaire built to provide a simple and generic measure of health.

**Results:**

Thirty-day mortality was 60.9%, and in-hospital mortality was 70.3% (*n* = 45). No patient was discharged with a stoma. Patients with a primary anastomosis after the initial operation for AMI had a high leak rate of 27% (4/15 patients) compared to no dehiscence in the group of patients who had secondary anastomosis during second or third laparotomy. The long-term survivors had significantly lower CCI compared to the 45 nonsurvivors (median 4 [3, 4, 5, 6] vs. 6 [4, 5, 6, 7]). All long-term survivors had QoL assessment. QoL score was significantly impaired compared to an age- and sex-matched reference population. This impairment was not due to disease-specific sequelae such as presence of stool deviation or intestinal failure but due to preexisting risk factors as shown by an inverse relation between the CCI and QoL score.

**Conclusion:**

Herein, we show for the first time that long-term QoL in patients with AMI is impaired but this impairment is not due to disease-specific aspects but rather general risk factors underlying the presence of a higher level of comorbidities at the time of AMI.

## Introduction

The dilemma with acute mesenteric ischemia (AMI) is based on the fact that the prognosis is not only poor but that it is also difficult to predict in the acute setting for the individual patient. Prognostic factors for irreversible bowel necrosis have been established [1], but early detection of AMI is still the clue to successful outcome [2]. Studies reporting on AMI are exclusively retrospective [3] making it difficult to establish evidence-based guidelines [4–6]. Efforts have been made to summarize recent data for meta-analysis in view of the best therapeutic approaches comparing interventional with open revascularization techniques [7, 8]. A dedicated multidisciplinary team as for ischemic stroke has been shown to improve survival [9, 10].

Age, BMI, ASA, number surgeries, total length of bowel resected, length of stay, and number of comorbidities are given as median with 25–75 percentile. Dunn's test for multiple comparisons between groups with * *p* < 0.05 between venous and embolic or thrombotic.

AMI predominantly occurs in the elderly population having relevant comorbidities. It is estimated that 25% of patients with embolic AMI already take oral anticoagulations [11]. Thrombotic AMI reflects general vasosclerosis, with concomitant cardiac and renal comorbidities. Only venous mesenteric occlusive disease usually affects younger, predominantly male patients having fewer comorbidities [12, 13]. Studies addressing risk factors for mortality in AMI have mainly investigated the role of single factors [2, 14]. From a clinical viewpoint however, the sum of comorbidities may rather determine prognosis. As such, the Charlson comorbidity index (CCI) was introduced to assess the role of comorbidities for overall prognosis [15] and is nowadays also used to predict outcome after surgery [16, 17]. Few studies have investigated the CCI as predictor of outcome for AMI with divergent results [18, 19].

Quality of life (QoL) has become a relevant outcome parameter for many clinical entities such as chronic inflammatory bowel disease or rectal cancer [20]. In some cases, QoL is determined by the disease itself and in some cases by the therapy such as in low anterior resection syndrome after multimodal therapy for rectal cancer [21]. Specific impairment of QoL after AMI could be due to having a stoma or to intestinal failure necessitating parenteral nutrition. Whether patients with AMI display impaired QoL after the emergency event can be suspected but has not been analyzed in detail since most studies focus on acute outcome parameters mainly survival. The EQ-5D is a simple but validated instrument to subjectively assess QoL. Country-specific reference populations stratified by age and gender have been established making them suitable for comparison of an experimental cohort [22, 23]. In this retrospective study, we assessed short- and long-term outcome after AMI and put the focus on QoL in the long-term survivors by correlating impaired QoL with preexisting comorbidities.

## Material and Methods

All patients with AMI who were treated in the department of general, visceral, vascular, thoracic, and transplant surgery of the University Medical Center Rostock between January 1, 2008, and December 31, 2016, were included in this retrospective study. Patients were identified in the hospital documentation system using specific OPS codes indicating a procedure related to AMI. Additional data were gathered by chart review. Patients with nonocclusive mesenteric ischemia were excluded. 64 patients were finally included in the analysis. The study was approved by the Rostock University Medical Center Ethics Committee (A2022-0049).

For long-term follow-up, patients were contacted and interviewed between July and October 2017 using a custom-made questionnaire and the EQ-5D health questionnaire including the EQ-5D visual analog scale (VAS) [24]. In the EQ-5D mobility, self-care, usual activities, pain/discomfort, and anxiety/depression are rated at 5 levels (none, slight, moderate, severe, and extreme). The resulting QoL and the patient-reported EQ-5D VAS are adjusted to age, sex, and a German reference population [22, 25]. QoL data were compared using the normalized *t* test assuming that the reference population has a value of “1.”

The CCI was calculated retrospectively using the age of the patient and the documented comorbidities at the time of surgery [15]. Statistical analysis was performed using GraphPad Prism^®^ version 8.4.3. Results are represented as median with 25–75 percentiles unless otherwise indicated. Data were checked for normality and then compared by the appropriate test as stated in the text. Simple linear regression was used for correlation between the points of the CCI and the QoL score as measured by EQ-5D.

## Results

### Short-Term Results

Sixty-four patients were included in the analysis. Fifty-six patients had acute arterial occlusive mesenteric ischemia of whom 36 had embolic and 20 thrombotic occlusion. Eight patients had acute venous occlusive ischemia. There was no difference in BMI, total length of resected bowel, or number of surgical interventions between the groups. However, age, ASA score, and CCI were significantly lower in the venous occlusive group compared to both arterial groups (Table 1).

We chose diabetes, arterial hypertension, atrial fibrillation, and chronic renal insufficiency as risk factors for AMI. As expected, patients with arterial mesenteric ischemia (embolic or thrombotic) had significantly more risk factors compared to patients with venous occlusive ischemia (Table 1). At the time of the acute episode, only 19% of the patients with embolic occlusion were on oral anticoagulation but 65% of patients with atherosclerotic disease were receiving antiplatelet therapy.

**Fig. 1:**
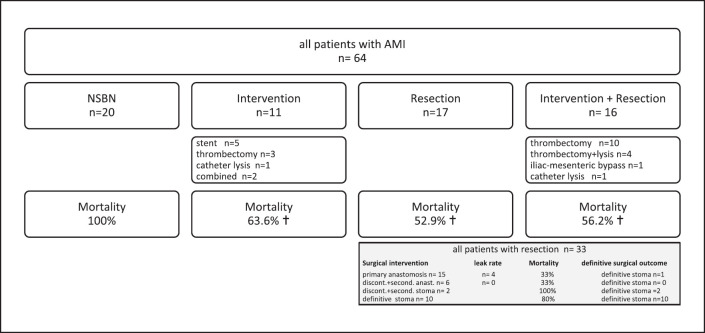
Operative procedures and interventions. Nonsurvival bowel necrosis (NSBN) at laparotomy resulted in 100% mortality. Mortality in each treatment group was significantly lower compared to the NSBN group (*p* < 0.05 by Kruskal-Wallis test). Patients with bowel resection (gray-shadowed box) (*n* = 33) were divided into 4 subgroups: resection with primary anastomosis, resection with discontinuity and secondary anastomosis or secondary stoma, and resection with definitive stoma. Dehiscence rate and mortality as well as the rate of definitive stoma for each of these four groups are listed in rows.

Twenty patients were explored by laparotomy or laparoscopy and showed nonsurvival bowel necrosis (NSBN) resulting in 100% mortality. Eleven patients were explored and had vascular intervention but no bowel resection, 17 patients had bowel resection only, and 16 patients had both vascular intervention and bowel resection. Thrombectomy was performed either by the surgeon or immediately after surgery by the interventional radiologist. It was the most commonly performed procedure followed by stenting of the superior mesenteric artery or local lysis via a catheter in the superior mesenteric artery. One patient received an iliac-mesenteric bypass (Fig. 1).

Surgical procedures in the group of patients who experienced bowel resection are summarized in the shaded gray box in Figure 1. Fifteen patients received a primary anastomosis after bowel resection of whom 4 showed anastomotic dehiscence resulting however in only 1 definitive stoma. Eight patients had bowel resection at the first operation leading to temporary discontinuity of the gastrointestinal tract. Of these, 6 had secondary anastomosis and 2 had a stoma created at the second operation. There was no leakage in this group of patients who received secondary anastomosis (*n* = 6). Ten patients had bowel resection with definitive stoma, and mortality in this group was 80%.

Thirty-day mortality of the total cohort was 60.9% (*n* = 39), and hospital mortality was 70.3% (*n* = 45). Nonsurvivors had a significant higher CCI compared to survivors (6 [4–6] vs. 4 [3–6], *p* < 0.05). There was a strong trend to significance for the length of resected bowel between survivors and nonsurvivors (*p* = 0.052). Age however was not different between both groups (Table 2). Patients with arterial occlusive ischemia had significantly lower survival compared to patients with venous occlusive ischemia (*p* < 0.006, by Fisher's exact test).

## Long-Term Results

Of the 19 patients discharged after AMI, 7 patients died before follow-up resulting in 12 patients (19%) evaluable as long-term survivors. The median follow-up of these 12 patients was 1,482 days (range 504–3,334). Seven of the long-term survivors were in the arterial group and 5 in the venous group. One patient had signs of short bowel syndrome which was orally compensated, and none of the patients received long-term parenteral nutrition. Three patients had an incisional hernia, and 1 patient had needed reoperation due to adhesions. None of the long-term survivors had a stoma.

**Fig. 2:**
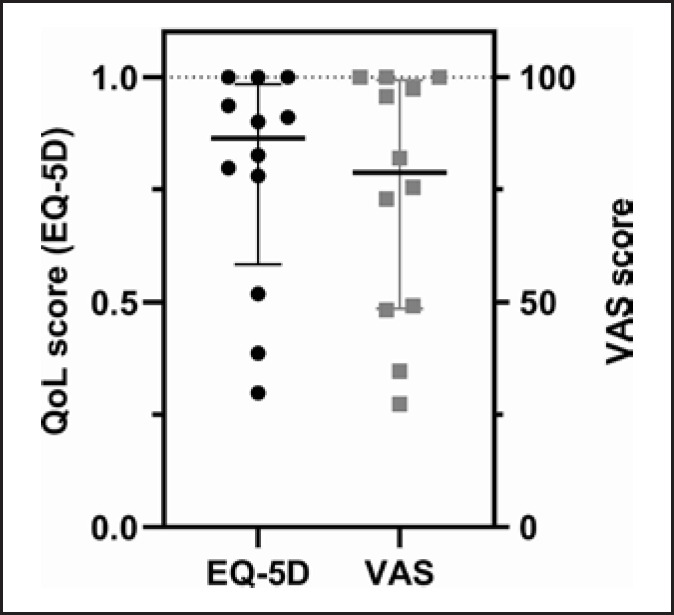
QoL measured by the EQ-5D is graphically displayed by the QoL score (left side) which represents the weighted sum of 5 separately evaluated parameters. One is the maximum value and represents “best health.” The VAS (right side) is a patient-reported self-estimation where 100 represents the maximum. The QoL score of the long-term survivors is significantly lower compared to the reference population (*p* < 0.0039 by one sample Wilcoxon test).

The long-term survivors (*n* = 12) had a significantly lower CCI (3.5 [0.25–5] vs. 6 [4–7]) and significantly fewer risk factors (median 0.5 [0–2] vs. 2 [1–3], *p* < 0.031 by Mann-Whitney U test) compared to the 45 nonsurvivors indicating that the CCI and the number of risk factors determine short-term outcome. Long-term survivors had a significantly diminished QoL score as measured by EQ-5D and expressed relative to a reference population which is set as “1.” The self-estimated VAS was also numerically lower, but this did not reach statistical significance (Fig. 2). There was no difference in QoL score between the arterial and the venous groups (not shown); however, the CCI was significantly lower in the venous group (0 [0–2] vs. 5 [4–6], *p* < 0.05). The length of the resected bowel correlated with impairment of QoL (*p* < 0.05 by linear regression).

**Fig. 3:**
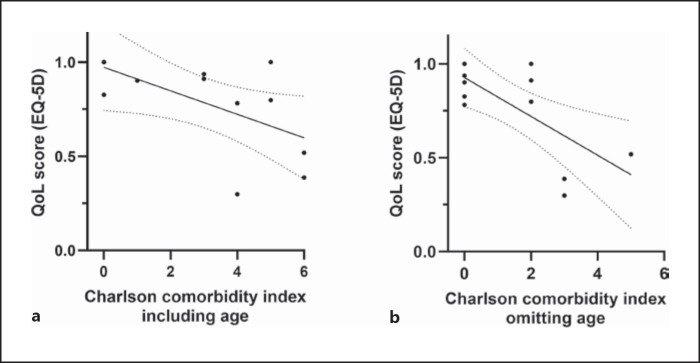
Correlation of the Charlson comorbidity index (CCI) with the QoL score represented by the EQ-5D. In **a,** the linear regression between the CCI and QoL score is shown. In **b,** the Charlson comorbidity index (CCI) excluding age as one risk factor is correlated with the QoL score. In **a** and **b,** there is a significant inverse correlation between the CCI and QoL score (*p* < 0.05 by Spearman test).

There was a significant inverse correlation between the CCI and the self-reported QoL score indicating that a higher CCI correlates with a lower QoL (Fig. 3a). Age is one of 17 parameters composing the CCI. Age attributes to the CCI with one point for each decade starting at the age of 50 years. Since in our cohort age is a strong contributor to a higher CCI especially in the arterial group but age was not different between survivors and nonsurvivors, the linear regression analysis was repeated omitting age in the CCI calculation. The results still show a significant inverse correlation (Fig. 3b). Taken together, these results suggest that the impaired QoL in patients with AMI is due to their underlying comorbidities but not to age and not to the sequelae of the acute event.

## Discussion

The data of this single-center cohort of 64 patients with occlusive mesenteric ischemia underline the poor prognosis of this disease with a 30-day mortality of 61% and a hospital mortality of 70% going along with a recently published study [11]. Of note, age was not significantly different between survivors and nonsurvivors but this may be due to the small size of the cohort. Since our cohort included 8 patients with venous occlusion disease of whom 5 survived until follow-up, prognosis for arterial occlusion alone is even worse [14].

Almost a third of the cohort (*n* = 20) had NSBN, and only half (*n* = 33) had bowel resection. As shown previously [26], patients who had a two-step approach with reanastomosis after initial discontinuity resection had a better outcome and a lower leak rate than patients with primary anastomosis. We therefore judge secondary anastomosis without temporary stoma as “safe” and apply this concept in general in the acute setting providing the patient with a good chance to stay without an enterostomy. The presence of a stoma might be a determining factor for QoL especially in the elderly patient. EQ-5D is a validated tool of general QoL without special focus on gastrointestinal symptoms. We specifically chose an QoL instrument which measures general health of this elderly population.

Whether treatment consisted of resection only or the combination of vascular intervention with resection did not influence mortality in our cohort. Recent publications point toward a benefit of endovascular therapy over surgical revascularization [27] not only in regard to mortality but also in regard to bowel resection [7]. In our cohort as well as in others [10], the length of the resected bowel correlates with outcome. It can be speculated that this is one reason for the absence of intestinal failure in our cohort. Although we did not address this specifically, length of resection is usually related to central versus peripheral occlusion.

Twelve of the 19 survivors with a median follow-up of about 4 years were included in the QoL analysis. The EQ-5D is a simple and well-validated tool to measure QoL by addressing five parameters, namely, mobility, self-care, usual activities, pain/discomfort, and anxiety/depression. The EQ-5D is an ideal questionnaire in this elderly patient cohort since it adjusts QoL for age and gender which are important aspects.

AMI is an acute disease which poses the patient as well as the physician into a situation where rapid decision is crucial. Since time to treatment start determines outcome, exhausting reflection about treatment options is not applicable. Patients however need to have basic information about their probable outcome in terms of short-term survival and long-term QoL. Data about QoL after acute occlusive mesenteric ischemia are missing. Only one publication exists for QoL after intervention in chronic mesenteric ischemia [28]. To the best of our knowledge, this study reports for the first time QoL data in AMI survivors with a median follow-up of 4 years. We conclude from our data that the lower QoL is not due to AMI-related sequelae such as parenteral nutrition or presence of an enterostomy since none of these complications were present at follow-up. There is however an inverse correlation between the CCI with QoL score indicating that not a single factor determines QoL but rather the sum of comorbidities at the time of AMI. In our cohort, age contributed to a higher CCI especially in the arterial group. By recalculating the CCI without age however, the inverse correlation of the CCI with QoL score remained unchanged indicating that age at the time of AMI does not determine long-term QoL. Age should therefore not be considered as a limiting risk factor when considering treatment options.

## Conclusion

In summary, in this cohort of 64 patients assembled over a 9-year period with occlusive mesenteric ischemia, we show for the first time that QoL is impaired compared to a reference population. This impairment is not due to specific ischemia-associated sequelae such as living with an enterostomy or other gastrointestinal symptoms but is rather reflected by the extend of comorbidities present in the individual patient at the time of surgery.

## Statement of Ethics

The study was conducted in accordance with the ethical principles outlined in the Declaration of Helsinki. This study protocol was reviewed and approved by the Rostock University Medical Center Ethics Committee, including an exception of obtaining written informed consent to participate (oral consent was obtained instead), approval number (A2022-0049).

## Conflict of Interest Statement

The authors have no conflicts of interest to declare.

## Funding Sources

None to declare.

## Author Contributions

Conception and design: Manuela Neese, Maria Witte, and Mark Philipp; collection and assembly of data: Manuela Neese and Maria Witte; statistical analysis: Maria Witte and Matthias Leuchter; data interpretation: all authors; manuscript writing: Maria Witte; final approval of the article: all authors.

## Data Availability Statement

All data generated or analyzed during this study are included in this article. Further inquiries can be directed to the corresponding author.

## Figures and Tables

**Table 1:** Demographic and clinical data of the patient cohort with AMI stratified by type of AMI

	Total	Arterial		Venous	*p* value
		embolic	thrombotic		
Number	64	36	20	8	
Sex female/male	33/31	21/15	9/11	3/5	
Age, years	71 (62–79)	73.5* (68–82)	69 (61–74)	51.5 (47–72)*	<0.012
BMI	26 (23–30)	27.6 (24–33)	24.8 (21–28)	26.3 (22–28)	ns
ASA	4	4*	4*	3*	<0.02
Number of surgeries, *n*	2 (1–3)	2 (1–3)	1 (1–3)	3.5 (1–5)	ns
Length of resected bowel, cm	110 (53–180)	110 (70–190)	170 (40–210)	60 (45–80)	
CCI	5 (4–7)	6 (4–7)*	5.5 (4–7.5)*	2 (0–4.5)*	<0.05
Risk factors (*n*/patient)	2 (1–3)	2 (2–3)*	1.5 (1–2.75)	0 (0–1.5)*	<0.045
Diabetes, %	30	36	35	12.5	
Hypertension, %	66	75	80	25	
Arterial fibrillation, %	44	75	15	0	
Renal insufficiency, %	33	36	45	12.5	
Anticoagulation, %	14	19	5	12	
Antiplatelet therapy, %	44	44	65	0	

Age, BMI, ASA, number surgeries, total length of bowel resected, length of stay, and number of comorbidities are given as median with 25–75 percentile. Dunn's test for multiple comparisons between groups with

* *p* < 0.05 between venous and embolic or thrombotic.

**Table 2:** Parameters of short-term outcome relevant for long-term QoL

	Nonsurvivors (*n* = 45)	Survivors (*n* = 19)	*p* value
Age	73 (65.5–79)	70 (52–74)	ns
Length of resected bowel, cm	130 (77–195)	80 (40–170)	0.052
CCI	6 (4–7)	4 (3–6)	0.017


Age, length of resected bowel, and CCI are given as median with 25–75 percentile. Comparison by Mann-Whitney test.
